# Rare Case of Ethmoidal Encephalocele and Sequelae

**DOI:** 10.5811/cpcem.48554

**Published:** 2026-02-24

**Authors:** Kiveum Kim, Taylor Craig, Lucas Delicio, Alexander John Scumpia

**Affiliations:** HCA Florida Aventura Hospital, Department of Emergency Medicine, Aventura, Florida

**Keywords:** basal encephalocele, atraumatic, primary (congenital) encephalocele, atraumatic encephalocele

## Abstract

**Case Presentation:**

A 64-year-old Black female presented to the emergency department following a new-onset tonic-clonic seizure. The patient had been given 2 milligrams of lorazepam by emergency medical services with cessation of seizure activity. On physical exam she was lethargic and had clear discharge from the right nare. Computed tomography of the brain initially demonstrated findings consistent with sinusitis versus ethmoidal mass. Magnetic resonance imaging of the brain demonstrated a right frontal ethmoidal encephalocele.

**Discussion:**

Basal encephaloceles occur due to a defect in the skull base. Location of the defect and extracranial herniation of brain tissue can cause neurologic sequelae. This case illustrates the importance of maintaining a broad differential diagnosis and for emergency physicians to obtain imaging when evaluating seizures and/or chronic rhinorrhea in adults.

## CASE PRESENTATION

A 64-year-old Black female presented to the emergency department following a new-onset tonic-clonic seizure. The patient endorsed chronic rhinorrhea from the right nare intermittently for 25 years and increased frequency of rhinorrhea over that time frame, but she denied acute changes. No alleviating or exacerbating factors were associated with her rhinorrhea. On physical exam, the patient had clear nasal discharge from the right nare, with otherwise normal facial features. She initially presented post-ictal, but after returning to her baseline she had no focal neurological deficits, with cranial nerves II–XII grossly intact. Magnetic resonance imaging of the brain demonstrated an ethmoidal encephalocele (Images A and B). Beta-2 transferrin testing of rhinorrhea was performed, confirming cerebrospinal fluid leak.

The patient was admitted and evaluated by neurology. Subsequent electroencephalogram demonstrated diffuse encephalopathy consistent with post-ictal state with right frontal encephalocele assumed to be the epileptic focus. She was started on phenytoin and subsequently discharged with instructions to follow up with neurosurgery. After undergoing endoscopic repair, a six-month follow-up deemed the repair successful and she was symptom-free. The patient was instructed to stop taking phenytoin. The patient has had no subsequent seizures post-resection of the anterior cranial fossa encephalocele.

## DISCUSSION

Basal encephalocele is a type of primary (congenital) encephaloceles that occur due to a primary defect in the skull base. They can be further classified based on the location of the defect. A defect in the dura mater permits the extracranial herniation of brain tissue, and subsequent leptomeninges and cerebrospinal fluid into the extracranial space. The pathogenesis is believed to be a defective closure of the anterior portion of the neural tube early in the developmental process.^1^ Consequently, basal encephaloceles often present with other clinical features of patients with neural tube defects including broadened nasal bridge, hypertelorism, and other midfacial anomalies.^2^ They are primarily diagnosed in the prenatal period with serum alpha fetoprotein levels and ultrasonography or early in childhood in patients who have associated phenotypic facial features.

It is much more difficult to diagnose primary encephalocele in adults, especially in those who lack the classical facial features, as was the case with our patient.^3^ Secondary (acquired) encephalocele is associated with cranial defects from injuries, such as those that occur in post-traumatic or post-surgical patients. The lack of associated facial features common in congenital encephalocele along with a lack of the classic presentation (post-traumatic and post-surgical history) made this a unique presentation and difficult to diagnose. Once the diagnosis is made, prompt treatment is critical to prevent complications including meningitis, tension pneumocephalus, and seizures.^4^ Treatment includes resection of the anterior cranial fossa encephalocele.^5^

*CPC-EM Capsule*

What do we already know about this clinical entity?

*Basal encephaloceles are rare congenital skull base defects causing cerebral spinal fluid (CSF) leaks, seizures, or meningitis, usually diagnosed in infancy*.

What is the major impact of the image(s)?

*The magnetic resonance images confirm ethmoidal encephalocele and CSF leak, distinguishing it from sinus disease and guiding neurosurgical management*.

How might this improve emergency medicine practice?

*Emergency clinicians should be aware that chronic rhinorrhea with seizures may signal encephalocele, prompting imaging and early referral*.

## Figures and Tables

**Image A:**
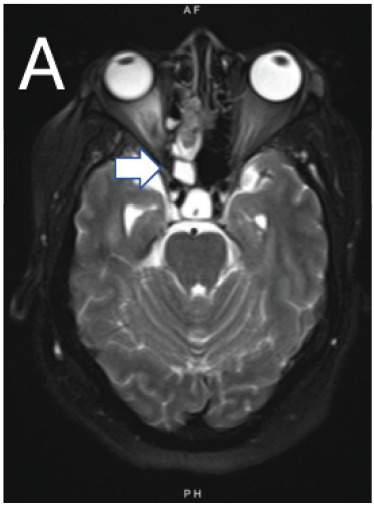
Axial T2-weighted magnetic resonance imaging of the brain demonstrating herniation of the right inferior frontal lobe tissue consistent with primary encephalocele (arrow).

**Image B:**
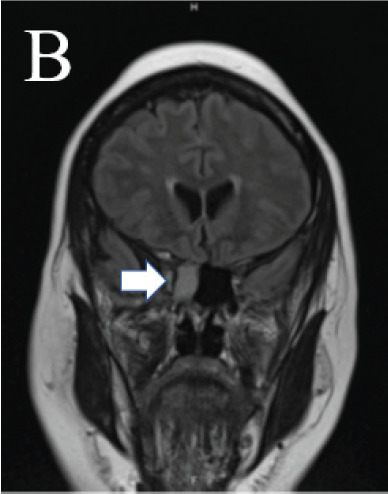
Coronal T1-weighted magnetic resonance imaging of the brain demonstrating herniation of the right inferior frontal lobe tissue through the right cribriform plate (arrow) into the sphenoid sinuses.
